# [Retracted] Activation of GPER by E2 promotes proliferation, invasion and migration of breast cancer cells by regulating the miR-124/CD151 pathway

**DOI:** 10.3892/ol.2025.15429

**Published:** 2025-12-15

**Authors:** Huaicheng Yang, Congyu Wang, Heqiang Liao, Qi Wang

Oncol Lett 21: 432, 2021; DOI: 10.3892/ol.2021.12693

Following the publication of this paper, it was drawn to the Editor's attention by a concerned reader that, for the cell invasion assay experiments shown in Fig. 3D on p. 5, in addition to the identification of a pair of overlapping data panels within this figure, some of the data were strikingly similar to data that had already appeared in another paper written by different authors at different research institutes that was published in the journal *Experimental and Therapeutic Medicine*.

Owing to the fact that the contentious data in the above article had already been published before this paper was submitted to *Oncology Letters*, the Editor has decided that this paper should be retracted from the Journal. After contacting the authors, they accepted the decision to retract the paper. The Editor apologizes to the readership for any inconvenience caused.

